# Beware of neonatal appendicitis

**DOI:** 10.4103/0971-9261.70646

**Published:** 2010

**Authors:** Rizwan A. Khan, Prema Menon, K. L. N. Rao

**Affiliations:** Department of Pediatric Surgery, PGIMER, Chandigarh, India

**Keywords:** Appendicitis, neonate, necrotizing enterocolitis, peritonitis

## Abstract

We report a neonate with acute appendicitis who was mistakenly diagnosed and treated initially as neonatal necrotizing enterocolitis. The diagnostic dilemma of this rare and life-threatening condition in premature babies and newborns is underlined. Awareness of this rare condition and possible differential diagnosis in this age group is also discussed.

## INTRODUCTION

The rarity of neonatal appendicitis together with lack of specific signs and low index of suspicion make it very difficult for the attending clinicians to make an early diagnosis.[1] Moreover, the chances of perforation are high leading to peritonitis causing high morbidity and mortality. Appendicular perforation, in this age group, may represent an underlying disease and therefore, Hirschsprung’s disease (HD), cystic fibrosis (CF), and isolated form of necrotizing enterocolitis limited to the appendix should always be ruled out.

## CASE REPORT

A 23-day-old full-term male weighing 2700 g, born by normal delivery to a second gravida mother with an uncomplicated perinatal history was brought to the pediatric emergency. The baby was apparently normal in the first 15 days of life, but when he refused to feed, he vomited and developed abdominal distension. After one week of treatment at a local hospital, the patient was referred to our center. On the eighth day of his illness, the patient had high fever, tachycardia, increasing abdominal distension, and increasing oxygen requirement. The physical examination revealed mild respiratory distress and a markedly distended, tender abdomen with no skin changes. The leukocyte count was 14,500/dL. The renal function tests were within normal limits. A diagnosis was made of peritonitis, possibly secondary to neonatal necrotizing enterocolitis (NNEC), and supportive treatment was started. On the 9^th^ day, plain radiographs of the abdomen showed free air in the peritoneal cavity, dilated ileal loops, some air–fluid levels in the lower abdomen but no evidence of pneumatosis intestinalis. After placement of a left-sided flank drain, the baby’s respiration improved. However within 24 h, small bowel prolapsed through the drain site and the patient had to be taken up for emergency laparotomy. The peritoneal cavity was contaminated with fibrinopurulent debris especially in the right lower quadrant. The appendix, measuring 3.0 cm in length was perforated near the tip. There were no obvious signs of NNEC, Hirschsprung’s disease or other obvious anomalies in the intestine. An appendicectomy was performed and full-thickness biopsies taken from multiple sites from the large bowel. Histologic examination of the appendix showed that the lumen was filled with concretions with dense fibrinosuppurative infiltrate and transmural granulation at the site of perforation. Normal ganglia were found in all specimens of the large bowel. The postoperative period was uneventful. The child was thriving at six-month follow-up and his milestones were normal.

## DISCUSSION

While acute appendicitis is a common occurrence in childhood, it is hardly ever considered in the differential diagnosis of acute abdomen in neonates. The incidence of neonatal appendicitis has been variously reported as 0.04 to 0.2% and is more frequent in premature males.[1–3] This low incidence is due to several factors. The appendix is still in its fetal form i.e. funnel shaped having wide opening into the cecum, and thus less prone to obstruction than the mature finger-like shape in older children.[3] Intraluminal obstruction is unlikely in neonates due to recumbent posture and liquid diet.[4]

In neonates, it is extremely difficult to properly assess the presentation leading to a delay in diagnosis. This leads to increased rate of perforation, peritonitis, and mortality. However some features are indicative. The baby may have irritability, distressed breathing, and wriggling indicating peritoneal inflammation. There may be abdominal distention, bilious vomiting with induration, edema and erythematous rash over the abdominal wall. This may be associated with swelling of the scrotum and a right lower quadrant mass. Other less consistent findings are anorexia, fever and leucocytosis. Abdominal radiograph may show abnormal gas pattern, free peritoneal fluid and air, thickened abdominal wall, a right scoliosis and obliteration of psoas margin. The presence of calcified appendicoliths, a radiographic presence seen in good number of patients in the older age group has never been reported in newborns.[5–7]

On abdominal ultrasonography, presence of intra-abdominal abscess, absence of gas in appendiceal lumen or evidence of collection in the right iliac fossa strongly suggests acute appendicitis. Spiral computed tomography can also be a very useful diagnostic tool.[8–10]

Perforation is a significant factor in determining the prognosis. As the signs and symptoms are not characteristic, the incidence of perforation is high in neonatal appendicitis. The other reasons are a thin appendiceal wall and an indistensible cecum.[6] A relatively small undeveloped and functionally non-existent omentum, small size of the peritoneal cavity allowing a more rapid and diffuse contamination and little physiological reserve, are important factors contributing to this high morbidity and mortality associated with perforation peritonitis in infants.[11]

However, contrary to the late childhood, perforation of appendix in this age group may be due to Hirschsprung’s disease, meconium plug syndrome, cystic fibrosis, necrotizing enterocolitis, and gastroenteritis.[12] These conditions cause obstruction or elevation of intracanalicular pressure in the lumen of the appendix. Since long-segment HD or total colonic aganglionosis usually cannot be excluded unless dilated and constricted zone can be seen clearly at operation, it may present as an appendiceal perforation in the neonatal period. In fact, the disease may be first diagnosed only years after an appendectomy is performed in the neonatal period for an apparent appendicitis. In Hirschsprung’s disease, the histology characteristically reveals periappendicitic changes, without mucosal involvement. In contrast, simple appendicitis shows evidence of panappendicitis. Therefore, it is crucial to get histological assessment of the resected specimen and if possible it should be supplemented with rectal and colonic biopsies.[12,13]

In cystic fibrosis, although the most common system to be affected is the respiratory system, abdominal complications including appendicitis can also be observed.[14] However, since these patients are usually on antibiotics for their respiratory illnesses, appendicitis may be missed. Therefore it is very important in these cases to get the histologic assessment of the appendix as it shows characteristic evidence of CF even in neonates.[15]

In the great majority of cases, acute appendicitis is rarely diagnosed preoperatively and is discovered only on postmortem examination. In this age group, symptoms such as irritability, drowsiness, nausea, vomiting, distension, and abdominal tenderness are not specific to appendicitis and can be found in myriad other abdominal conditions including generalized septicemia. In conclusion, judicious analysis of clinical symptoms and imaging findings and a high index of suspicion and the fact that the appendicitis should also be considered in the differential diagnosis with related clinical conditions can lead to early diagnosis and more timely surgical intervention to reduce the mortality of neonatal appendicitis.
